# Challenges of translating Arabidopsis insights into crops

**DOI:** 10.1093/plcell/koaf059

**Published:** 2025-04-03

**Authors:** Cristóbal Uauy, Hilde Nelissen, Raquel Lía Chan, Johnathan A Napier, David Seung, Linsan Liu, Sarah M McKim

**Affiliations:** John Innes Centre, Norwich Research Park, Norwich NR4 7UH, UK; Department of Plant Biotechnology and Bioinformatics, Ghent University, Ghent B-9052, Belgium; Center for Plant Systems Biology, VIB, Ghent B-9052, Belgium; Instituto de Agrobiotecnología del Litoral, Universidad Nacional del Litoral—CONICET, Facultad de Bioquímica y Ciencias Biológicas, 3000 Santa Fe, Argentina; Rothamsted Research, Harpenden, Herts AL5 2JQ, UK; John Innes Centre, Norwich Research Park, Norwich NR4 7UH, UK; Division of Plant Sciences, School of Life Sciences, University of Dundee, Dundee DD2 5DA, UK; Division of Plant Sciences, School of Life Sciences, University of Dundee, Dundee DD2 5DA, UK

## Abstract

The significance of research conducted on *Arabidopsis thaliana* cannot be overstated. This focus issue showcases how insights from Arabidopsis have opened new areas of biology and directly advanced our understanding of crops. Here, experts intimately involved in bridging between Arabidopsis and crops share their perspectives on the challenges and opportunities for translation. First, we examine the translatability of genetic modules from Arabidopsis into maize, emphasizing the need to publish well-executed translational experiments, regardless of outcome. Second, we highlight the landmark success of HB4, the first GM wheat cultivar on the market, whose abiotic tolerance is borne from direct translation and based on strategies first outlined in Arabidopsis. Third, we discuss the decades-long journey to engineer oilseed crops capable of producing omega-3 fish oils, with Arabidopsis serving as a critical intermediary. Fourth, we explore how direct translation of starch synthesizing proteins characterized in Arabidopsis helped uncover novel mechanisms and functions in crops, with potential valuable applications. Finally, we illustrate how shared molecular factors between Arabidopsis and barley exhibit distinct molecular wiring as exemplified in cuticular and stomatal development. Together, these vignettes underscore the pivotal role of Arabidopsis as a foundational model plant while highlighting the challenges of translating discoveries into field-ready, commercial cultivars with enhanced knowledge-based traits.

## Introduction

(Written by Cristóbal Uauy, editor)

*Arabidopsis thaliana* remains a remarkably powerful model plant species for gene discovery and mechanistic insights, providing a crucial foundation for hypothesis generation and testing in crops. However, as outlined in the vignettes and across the Focus Issue, translating insights from Arabidopsis to crops is often challenging due to differences in anatomy, physiology, genetics, and the limited tools available for functional characterization in many crop species. For instance, while research in Arabidopsis has deepened our understanding of epidermal development, differences and novelties in epidermal traits of grasses such as barley, including cuticle metabolism and stomatal organization, complicate direct translation. Similarly, natural variation in starch morphology across grasses, along with the diverse sites of starch synthesis, offers a natural “playground” to explore novel mechanisms for proteins initially identified in Arabidopsis. Reviews published across the Focus Issue highlight additional examples of translations related to abiotic interaction, plant-microbe interactions, plant immunity, and eco-evolutionary research.

Recent advances, such as the ability to transform commercial cultivars in many crops, are beginning to overcome long-standing bottlenecks, enabling researchers to investigate gene function in elite, commercially relevant genotypes rather than outdated varieties. As Raquel Chan aptly notes, improving an outdated variety is one thing—enhancing an elite cultivar under real-world field conditions is a far greater challenge. Translational work is inherently long term, unpredictable, and fraught with setbacks, from the laboratory to the field. These translational challenges tend to grow with increasing evolutionary distance, making it progressively more complex to translate findings first within dicots and then across to monocot species. Nevertheless, as Hilde Nelissen emphasizes, documenting these efforts—whether successful or not—is essential for progress.

Despite the challenges, notable success stories are emerging, such as drought- and heat-tolerant wheat and soybean, as well as oilseed crops engineered to produce omega-3 fish oils. These achievements underscore the importance of sustained commitment, interdisciplinary collaboration, and navigating complex regulatory frameworks. We hope these vignettes, along with the companion review by Adrienne Roeder and other articles in this Focus Issue, will inspire the plant science community to continue tackling the urgent need for sustainable food and nutritional security in the face of climate change.

## Let's turn a negative into a positive when interpreting translational research

(Written by Hilde Nelissen)

The extent of lack of trait transfer from Arabidopsis to crops has never been clearer than after an ag biotech company provided insights into their high-throughput translation efforts (Simmons et al. 2021). Even in our own research, where we transferred information about fundamental processes such as the regulation of cell division and cell expansion during organ formation from Arabidopsis to maize, the translational success rate was variable. Although cell division and cell expansion show remarkably similar spatial and temporal regulation in both Arabidopsis and maize (Nelissen et al. 2016), not all pathways identified in Arabidopsis (Vercruysse et al. 2021; Schneider et al. 2024) translate to maize.

**Figure 1.**
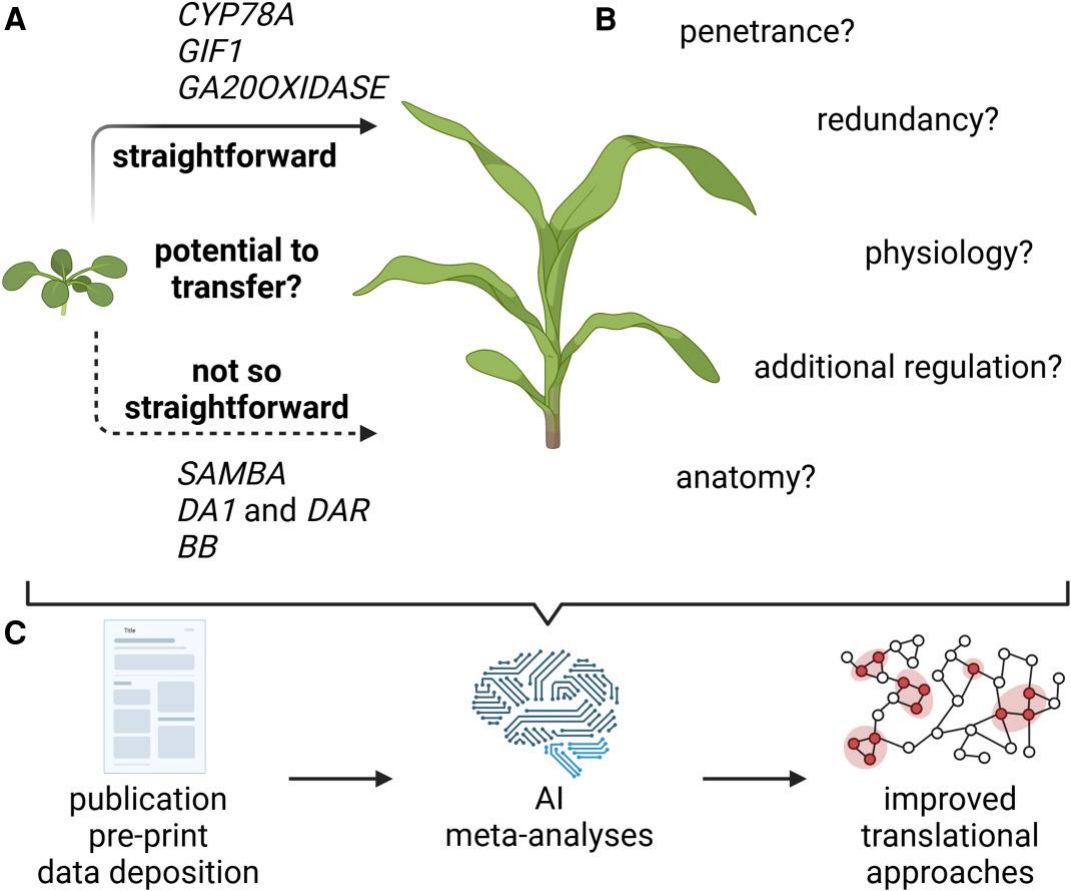
From translation to interpretation: growth processes as an example and recommendations for the future. **A)** In leaves, the growth processes of cell division and cell expansion are, to a certain extent, similarly regulated in dicots and monocots; for several pathways, orthologous genes are present in both Arabidopsis and maize. However, we currently lack an understanding of why similar genetic perturbations in Arabidopsis and maize sometimes lead to comparable phenotypic outputs (as seen with *GA20OXIDASE*, *CYP78A*, and *GIF1*), while in other cases they do not (e.g. *SAMBA*, *DA1*, *DAR*, and *BB*). **B)** Recognizing these “negative” translational outcomes and gaining insights into the potential reasons for these differences in “translatability”—such as differences in penetrance, physiology, anatomy, redundancy, or network rewiring—will be critical. **C)** Making data from well-executed experiments with lack of phenotypes available will serve meta-analyses and allow to incorporate this understanding into the design of future model-to-crop experiments to significantly enhance the ability to translate plant biotech research into practical applications. Created in https://BioRender.com.

Some pathways show a one-on-one translation (Fig. 1A) as is exemplified by modulating gibberellic acid levels that results in similar phenotypes in Arabidopsis and maize (González et al. 2010; Nelissen et al. 2012). Similarly, ectopic expression of *CYTOCHROME P45078A* family members increases organ size in both species (Anastasiou et al. 2007; Sun et al. 2017). However, translating findings across species can require multiple attempts. For example, overexpressing *GRF INTERACTING FACTOR1* (*GIF1*) in maize using the *ZmUBIQUITIN* promoter failed to replicate the Arabidopsis phenotype (Nelissen et al. 2015). Only with the *BdELONGATION FACTOR1alpha* promoter were phenotypes similar to Arabidopsis observed, highlighting the challenges of achieving sufficient expression levels in maize (Joossens et al. 2024). This importance of the correct timing, level, and location of expression illustrates the need to understand the spatio-temporal regulation and mechanistics of the processes as well as having access to the right tools to achieve the desired outcome, supporting the need for fundamental research in crops.

However, translation is not always as straightforward (Fig. 1A). For some growth-regulatory pathways, no clear homologs have been identified in grasses (Schneider et al. 2024), while others produce distinct phenotypes when perturbed. For instance, mutations in *SAMBA* in Arabidopsis enlarge leaves (Eloy et al. 2012), but in maize, they reduce leaf size (Gong et al. 2022a). This may reflect differences in cell cycle regulation, such as the absence of endoreduplication in maize. Similarly, Arabidopsis DA1 and DA1 RELATED proteases and the E3 ligase BIG BROTHER that activates them regulate organ size, but no comparable phenotypes were observed when their maize orthologs were similarly perturbed (Gong et al. 2022b). The difficulty of translating results across species stems from differences in physiology and anatomy or factors like regulatory differences, allele-specific effects, and genotype dependence in maize (Fig. 1B). The lack of phenotypes is often considered as a negative result, which is less interesting and impactful. However, these experiments often require more replicates and care to justify a conclusion that “under the given circumstances” the mutant or overexpression line did not deviate from wild type, rendering the results challenging to publish; this in turn discourages further exploration in maize.

Despite the reluctance of some journals to publish such “lost in translation” stories, these results of well-executed translation experiments that may not produce the desired result are urgently needed to better understand how we can more efficiently “translate” in the future. In the era of artificial intelligence, we need to obtain examples of straightforward translation as well as of neutral or even opposite phenotypic outcome as training sets to obtain reliable models for predicting translation efficiency. So as a plant community, we should invest in documenting translational research efforts. If we cannot make the data available via peer-reviewed journals or curated databases, we should at least make them available as preprints. This way, the data are available for meta-analyses on translatability, and we avoid investing research funds in replicating experiments (Fig. 1C).

In addition to improving the availability of results showing a translation bottleneck, we should also move away from considering these data as “negative” results. The cases in which translation does not lead to the expected result are often more interesting than the examples in which the “simple” 1-to-1 translation leads to the desired and comparable phenotype, because they raise many interesting biological questions as to why the translation is hampered. We should therefore avoid looking at translational research as a pure 1-to-1 translation and move to an interpretation where we consider more context, including the processes, molecular data, physiology, and anatomy (Fig. 1B). This interpretation is becoming more and more possible with the advent of pan-genomes (Gui et al. 2022), pan-cistromes (Engelhorn et al. 2023), single cell transcriptome atlases (Xu et al. 2024), and cross-species information (Curci et al. 2022). In addition, technological developments in crop plants allow for much more multiplex genome engineering to overcome redundancy (Lorenzo et al. 2023) and more genotype-independent transformation (McFarland and Kaeppler 2025).

The successful translation of insights from models to crops relies on a well-balanced integration of fundamental and applied research, as well as a synergy between model and crop studies. Arabidopsis research plays a central role in this process by uncovering mechanistic insights, driving technological advancements, and providing a foundational blueprint for genome annotations often extrapolated to other plant species (Yaschenko et al. 2024). However, it is essential to recognize that crop research within academia remains a form of fundamental science as well. Currently, the focus is shifting excessively toward application, with public funding agencies prioritizing crop research on elite germplasm, which is often tied to consolidated commercial markets. This emphasis places crop researchers in a challenging position, as selecting one variety over others is problematic for public funding purposes, and breeding companies are typically reluctant to allow sequencing and publication of their elite germplasm. Moreover, without genotype-independent transformation methods, introducing biotechnological innovations into elite varieties for field evaluations remains complex as well as time and resource intensive.

To foster innovation, a more practical approach is to conduct research on crop lines with lower economic value that are more amenable to transformation and for which substantial information is already available. Such efforts enable academic researchers to drive innovation, generate valuable insights, and provide actionable recommendations for breeders and companies. To ensure the continued advancement of plant science, it is therefore crucial to foster fundamental science and to maintain and strengthen the connections between Arabidopsis and crop researchers. This collaboration safeguards the innovative mission of publicly funded plant research and bridges the gap between fundamental discoveries and applied solutions.

## Why does translating abiotic stress tolerance from model plants to crops usually not have a happy end?

(Written by Raquel Lía Chan)

### Current situation of second-generation GMOs worldwide

Second-generation GMOs (genetically modified organisms) are almost absent from the market. It is sufficient to visit the web page of ISAAA (https://www.isaaa.org/), which presents all the information about approved transgenic events, to see dozens of herbicide- or insect-resistant crops, alone or combined and introduced in a variety of species, such as maize, alfalfa, cotton, and soybeans. However, for abiotic stress–tolerant or nutritionally enriched varieties, only a few events were approved, albeit mostly not commercialized (https://www.isaaa.org). The reasons are multiple, including the negative public perception and the prohibition of GMOs in many countries (Eriksson et al. 2020). However, the most important contributor is the lack of technologies that have proven efficient in field trials.

First-generation GMOs are easy to define: the crop resists or does not resist a given herbicide or insect, and there is no place for intermediary or fluctuating situations; such resistance does not depend on the environment. However, partial resistances were reported for certain evolving weeds involving mutations in genes encoding the protein targets of herbicides (Gaines et al. 2020). On the other hand, abiotic stress tolerance cannot be defined without considering the environment, changing every year, even for an individual region. A given improved crop could have a good performance in a place one year but not next year in the same or another place. This fact makes abiotic stress-tolerant crops less motivating for business (investments) than biotic stress-resistant ones (Chan et al. 2020). Hence, it is a great challenge for scientists to develop improved abiotic stress-tolerant crops exhibiting good performance in as many environments as possible.

### The main reasons explaining the lack of translated drought tolerance from Arabidopsis to crops

A quick search in PubMed using the term “drought tolerance” leads to more than 15,000 documents. Notably, when the searched term is “drought-resistance,” another 5,000 articles can be added to the list, even though there is no resistance to drought because all plants need at least some water to survive. Generally, such articles are published in prestigious journals describing the performance of a given overexpressed or silenced (by different methods) gene in various plant species. When adding “Arabidopsis” as a search term, the number of documents decreased to a little more than 3,000, still enormous. By choosing some articles at random, it is noteworthy that many are devoted to transcriptome analyses, lacking functional characterization. Others, assessing transgenic or mutant plants, did not include seed yield evaluation or were performed at the seedling stage. At the end of the abstracts, we usually find a sentence like “These results contribute to understanding the functions of the gene X, suggesting its potential in breeding stress-resilient crops.” Undoubtedly, it is valid to study a gene function under water deficit or another abiotic stress; the problem is the interpretation, with the terms drought resistance or drought tolerance often included in the title. That is because, in crop assessments, tolerance to any stress must be related to production (biomass, fruits, or grains, depending on the species). Without those data, farmers will not adopt such technologies.

It is hard to translate a given gene technology from Arabidopsis to crops and also from crop species grown in carefully controlled conditions to the field. Arabidopsis plants are grown in pots in carefully controlled conditions. Usually, a sole abiotic stress factor is applied in 1 experiment, maintaining optimal other growth conditions. In the field, plants live in a community, competing for light, nutrients, and water (Passioura 2010). It is impossible to control the conditions, and biotic and abiotic stressor factors are combined. Transcriptomes of plants subjected to combined stresses differ from the sum of the isolated transcriptomes (Mittler 2006; Rivero et al. 2014; Zandalinas et al. 2021; Pardo-Hernández et al. 2024). Hence, when adding the enormous differences between growth chambers or greenhouses and open fields to the fact that yield is rarely evaluated in such controlled experiments, the probability of failure is much greater than that of success. Another point to consider, being cautious in interpretations, is that it is easy to improve something of low quality. Most transformable crops are older, outdated varieties (Araus et al. 2019), as modern ones are not transformable. Doing several backcrosses with a better or commercial genotype leads to a modern variety crop bearing the overexpressed or edited gene. Hence, the differential beneficial traits observed in the first generation can be, partially or totally, lost in the subsequent generations.

### The story of HB4 wheat and soybean

*HaHB4* (*Helianthus annuus homeobox* 4) is a sunflower gene encoding a homeodomain-leucine zipper transcription factor. As in other cases, this gene was tested in Arabidopsis and found to confer water deficit tolerance (Dezar et al. 2005). Notably, this gene succeeded in improving soybean (27 field trials; Ribichich et al. 2020) and wheat (37 field trials; Waltz 2015; González et al. 2019) performance under drought and heat tolerance; *HaHB11*, another gene from the same family, also tested in Arabidopsis (Cabello et al. 2016), significantly improved seed yield in maize and rice plants (Raineri et al. 2022, 2023). Both soybeans and wheat transformed with *HaHB4* have been on the market since 2022, after a long pipeline of regulatory processes ending in safety approvals in many countries (https://www.isaaa.org; Miranda et al. 2022).

*HaHB11* crops are still undergoing the long regulatory processes. What was different about *HaHB4* and *HaHB11* compared with other genes in the literature? Besides good luck, the authors followed the paper published by the group of Dr. Dirk Inze (Skirycz et al. 2011), demonstrating that tolerance to severe drought was unrelated to seed yield in moderate or water-sufficient conditions. On the contrary, most drought-tolerant Arabidopsis genotypes exhibited seed yield penalties under well-watered or moderate stress conditions. This paper was the compass to evaluate each gene functionally in response to stress. As a rule, seed yield was assessed in many stress conditions, including moderate and severe stress, applied at different developmental stages. Once having promising results in such experiments, we can take the enormous risk of switching to crops, which is much more time-consuming and costly. And the same is true for gene editing. Undoubtedly, the second secret is the interdisciplinary approach, working with agronomists, who are experts in assessing field trials.

### The great challenge and future perspectives

**Figure 2.**
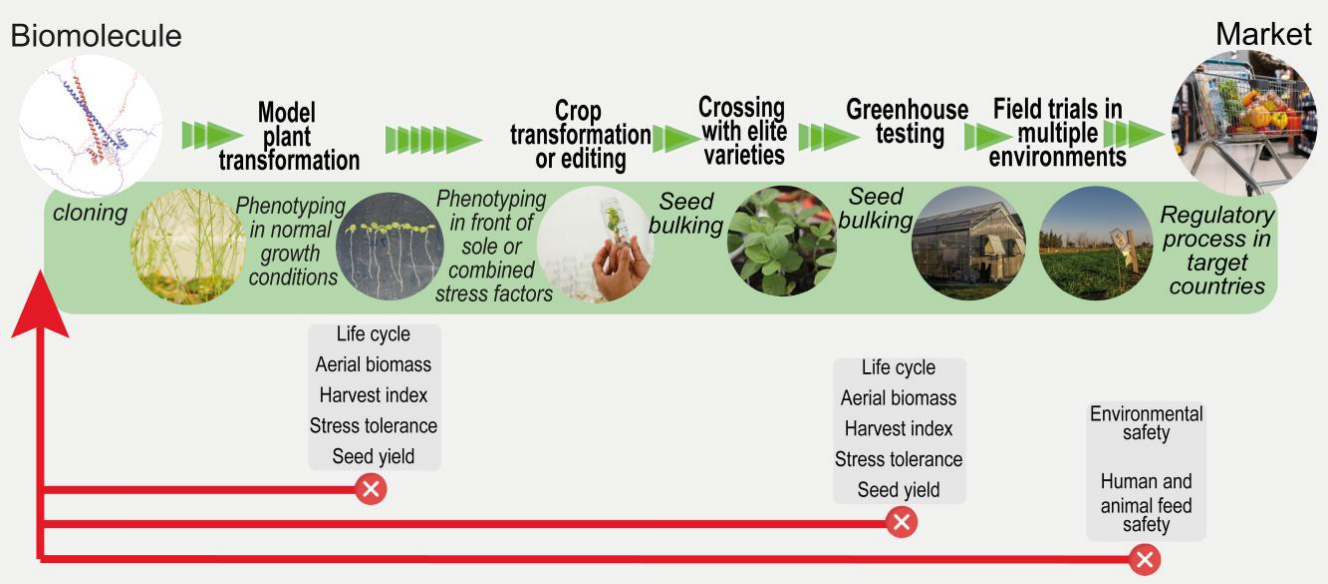
Schematic representation of the pipeline to follow to translate molecular technologies from Arabidopsis to crops. Schematic and abbreviated steps to be followed from gene discovery to arrive at a market product. Triangles indicate the way forward when there are beneficial effects. In the central square: illustrations of such steps and experiments needed to decide progress. The characteristics to be evaluated at each stage are shown in the bottom rectangles. Crosses for each characteristic indicate detrimental effects, determining the need to backtrack following the arrow.

HaHB4 is a successful example of translational research in plant molecular biology starting from the model plant Arabidopsis (Fig. 2; González et al. 2020; Gupta 2024). In this case, we can say that Arabidopsis undoubtedly did its job as a model system for gene discovery. For sure, it is not enough. Food security amid an increasing world population and climate change challenges requires more and more technologies to produce food in the same limited arable lands (Hall and Richards 2013). The challenge for plant scientists is to learn how to evaluate the performance of newly discovered genes to overexpress or mutate, to not overinterpret from experiments performed in model plants or in crops grown in controlled conditions, and, importantly, to work together with scientists from other disciplines to obtain a more holistic view of a given technology.

## *The Road Less Travelled*—converting a discovery into a useful product, as seen from the perspective of the Rothamsted omega-3 fish oil project

(Written by Johnathan A. Napier)

It is a well-appreciated fact that omega-3 long chain polyunsaturated fatty acids (LC-PUFAs; also known as omega-3 fish oils) are beneficial for human health, reducing the risk of cardiovascular disease and also playing key roles in neonatal development and wider anti-inflammatory responses (Choi and Calder 2024). Most vertebrates, including humans, have a very limited capacity to synthesize these fatty acids and therefore must obtain them from dietary sources. As the name implies, omega-3 fish oils are predominantly sourced from marine environments but unfortunately due to a combination of growing demand and over-exploitation, the fish stocks that provide these vital oils are at the maximum level of sustainability (Tocher et al. 2019). It was for this reason that over 25 years ago, myself and Olga Sayanova initiated a project to engineer plants with the capacity to synthesize the omega-3 LC-PUFAs eicosapentaenoic acid and docosahexanoic acid (EPA and DHA).

**Figure 3.**
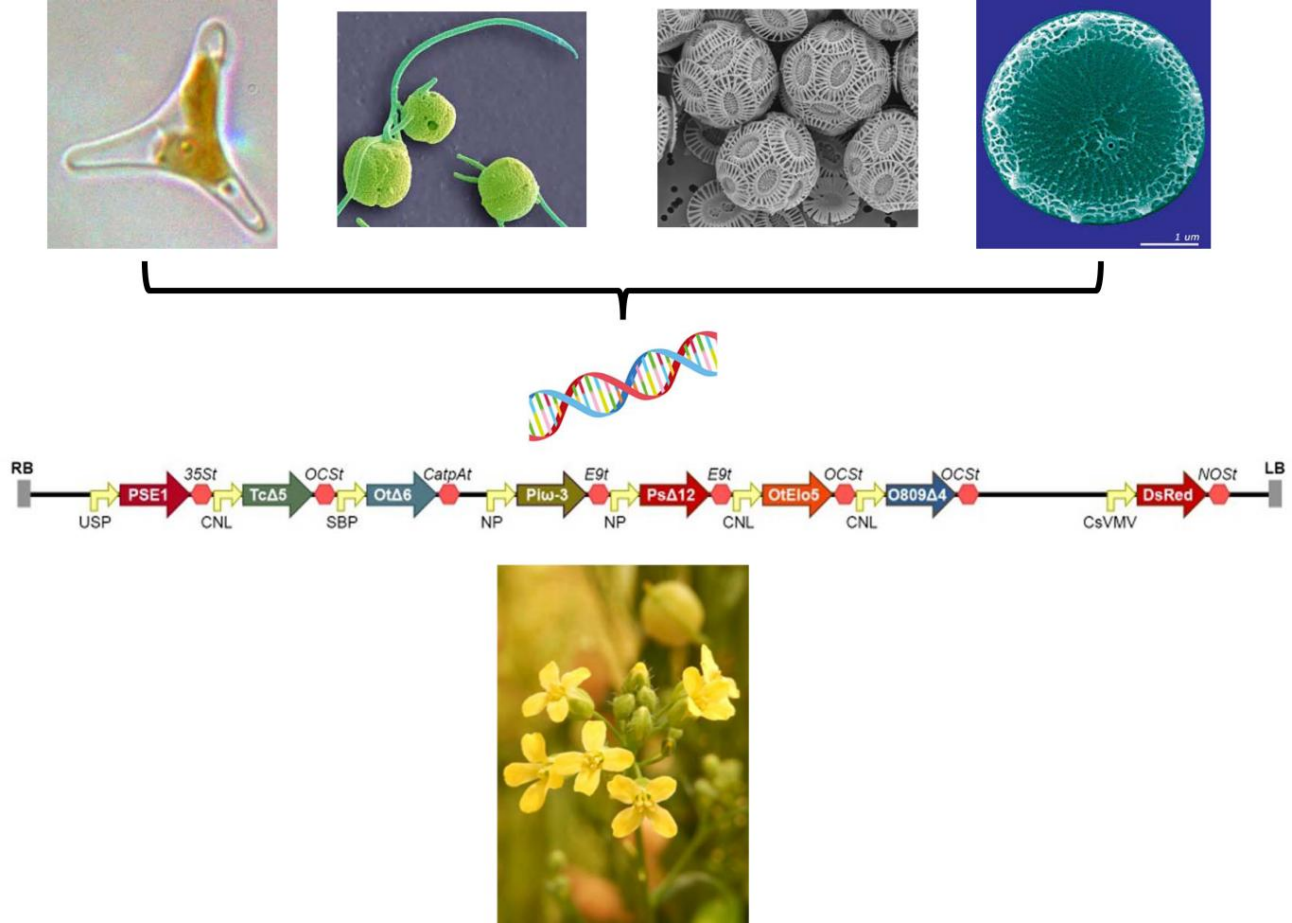
Schematic representation of engineering plants to accumulate omega-3 polyunsaturated fatty acids by the addition of genes from marine microalgae. Representative sources of biosynthetic genes are shown (from left to right: *Phaeodactylum tricornutum*; *Mantionella* spp.; *Emiliana huxleyi*; *Thalassiosira pseudonana*). Codon-optimized open reading frames encoding the enzymatic activities required for the synthesis of EPA and DHA were expressed under the control of seed-specific promoters and introduced into *Camelina sativa* by *Agrobacterium*-mediated transformation. The ability of Camelina to be transformed by floral dipping facilitated direct translation from studies in Arabidopsis.

The primary biosynthesis of EPA and DHA occurs at the base of the marine food web, being carried out by unicellular microalgae and bacteria, with these fatty acids accumulating in all higher trophic levels (Hamilton et al. 2020). The approach to engineer plants with the capacity to synthesize EPA + DHA is deceptively straightforward, requiring the transfer of genes from algae into a higher plant host (Sayanova and Napier 2004). However, several factors impeded initial attempts to reconstitute this pathway. These included the requirement for de novo gene discovery for the desaturases and elongases that generate EPA + DHA from endogenous precursors (Fig. 3A) along with only a partial understanding of the biochemical fluxes that underpinned this pathway. In addition, synthesis of EPA required a minimum of 3 genes and DHA required at least 5 genes, each under the control of their own (seed-specific) promoter (Venegas-Calerón et al. 2010). Thus, assembling these cassettes for expression in transgenic plants was technologically challenging in this pre-MoClo era (Fig. 3B). And although nowadays researchers are very familiar with the design-build-test-learn (DBTL) mantra of synthetic/engineering biology, this iterative methodology was crucial to the success of the omega-3 project (Ruiz-López et al. 2012).

Part of the success of the omega-3 project might be attributed to a pragmatic approach to advancing the research toward the ultimate goal of a viable prototype, suitable for scale-up and translation. The initial phase of gene discovery utilized simple systems like *S. cerevisiae* to validate individual biosynthetic activities and also to demonstrate the feasibility of reconstructing the PUFA pathway in a heterologous host (Beaudoin et al. 2000). However, since the ambition of this project was always to utilize the scale of agriculture, further advances focused on generating transgenic plants, initially in the model system Arabidopsis (on account of the ease of transformation). A landmark demonstration of the constitutive accumulation of EPA in Arabidopsis was published in 2004 (Qi et al. 2004), quickly followed by a related study demonstrating seed-specific accumulation in tobacco and linseed (Abbadi et al. 2004). Intriguingly, although the seed-specific expression of the biosynthetic genes (predominantly sourced from algae and also oomycetes and recoded for higher plants) achieved proof-of-principal accumulation of omega-3-LC-PUFAs (∼1–2% total fatty acids), non-native biosynthetic intermediates such as γ-linolenic acid and stearidonic acid accumulated to much higher levels (∼20% total fatty acids) (Abbadi et al. 2004). This metabolic bottleneck was subsequently shown to be due to the enzymes of the pathway (desaturases, elongases) working sequentially but utilizing different substrates. This process was termed substrate-dichotomy (Haslam et al. 2013) and was finally overcome by harmonizing the initial 2 steps of the pathway to use acyl-CoAs (Domergue et al. 2005). This allowed for the efficient, seed-specific accumulation of EPA and DHA in the seeds of transgenic plants. Importantly, it was shown that these non-native fatty acids accumulated in the seed oil triacylglycerols (Haslam et al. 2013). To date, the highest combined levels of EPA and DHA have been achieved in the Brassiciae *Camelina sativa* (Han et al. 2022), although significant levels of DHA alone have also been demonstrated in oilseed rape (Canola) (Petrie et al. 2020). In both cases, this was after initial iteration and validation in Arabidopsis, confirming the utility of this model system as a proof-of-principle workhorse for translational studies.

Creating a successful prototype transgenic plant accumulating high levels of EPA and DHA is a major achievement for plant biotechnology, demonstrating the potential for “green factories” to provide a sustainable alternative to the extraction of fish oils from marine stocks. But demonstrating the accumulation of these fatty acids in experimental plants grown under highly controlled laboratory conditions is very far from the real-world experience in the field and the gauntlet of biotic and abiotic challenges crops face in agricultural systems. Thus, the next essential step in validating this technology was to carry out field trials (Han et al. 2020), which served to not only confirm the robust nature of the EPA and DHA trait but also allow scale-up of seed, which in turn can be used to extract oil for use in de-risking studies demonstrating its efficacy as a drop-in replacement for fish oil in multiple applications (including aquaculture and direct human nutrition). Such translational studies also provide confidence for potential commercialization partners and would-be investors—in that respect, it is perhaps surprising (and slightly disappointing) how very few projects progress from the laboratory to the field (Ricroch et al. 2024)—within the plant sciences research community, there needs to be greater emphasis on field trials, at least for traits that are often articulated in funding pitches as delivering wider benefits beyond simple academic discovery. To that end, researchers and funders should both be more proactive in enabling positive research discoveries to move beyond the (literal and metaphorical) constraints of laboratory-based studies.

And although the step from laboratory to field is “the road less travelled” (Khaipho-Burch et al. 2023), it does not represent the end of the journey to bring an innovation to full utilization and impact—in the case of GM traits such as the omega-3 fish oils example described here, regulatory approval is necessary, for both commercial cultivation and use as a food or feed ingredient. Such approvals are usually obtained from national (as opposed to pan-national) agencies and in some jurisdictions can be both slow and costly to secure. A further issue that will be relevant to the commercialization of any trait is intellectual property, a topic that requires significant professional input.

Irrespective of all of these, the successful production of omega-3 fish oils in transgenic oilseeds and the subsequent translation and regulatory approval represent one of the best practical examples of the power of plant biotechnology to deliver to the twin goals of better nutrition and reduced environmental impact. Importantly, this was achieved through underpinning research carried out in model systems such as Arabidopsis and *S. cerevisiae*, leveraging knowledge and resources to drive discoveries forward to deliver genuinely useful innovations.

## Challenges in translation: starch synthesis in Arabidopsis vs crops

(Written by David Seung)

Despite not being starchy crops, Arabidopsis and other model systems (such as Chlamydomonas) have laid the foundation for developing a molecular understanding of starch synthesis (Ball and Morell 2003; Streb and Zeeman 2012). Starch is vital for human nutrition as a major dietary carbohydrate. It accumulates in large amounts in cereal grains and many root and tuber crops. Increasing starch yield is a major biotechnological goal, but it is equally important to improve its nutritional and functional quality to meet health and sustainability goals (Chen et al. 2021). For example, starch can be made less digestible in the gut to create more “resistant starch,” which acts as a fermentable dietary fiber that has positive impacts on gut microbiome and health (Li et al. 2019). Starch is also often modified using chemical and physical approaches to achieve the functionality required for manufacturing various food and nonfood products. These approaches can be energy intensive and environmentally harmful (Maniglia et al. 2021). Genetics offers an alternative method to achieve modified starch properties (Chen et al. 2021). Engineering such changes in starch requires a solid mechanistic understanding of its biosynthesis in crop plants.

**Figure 4.**
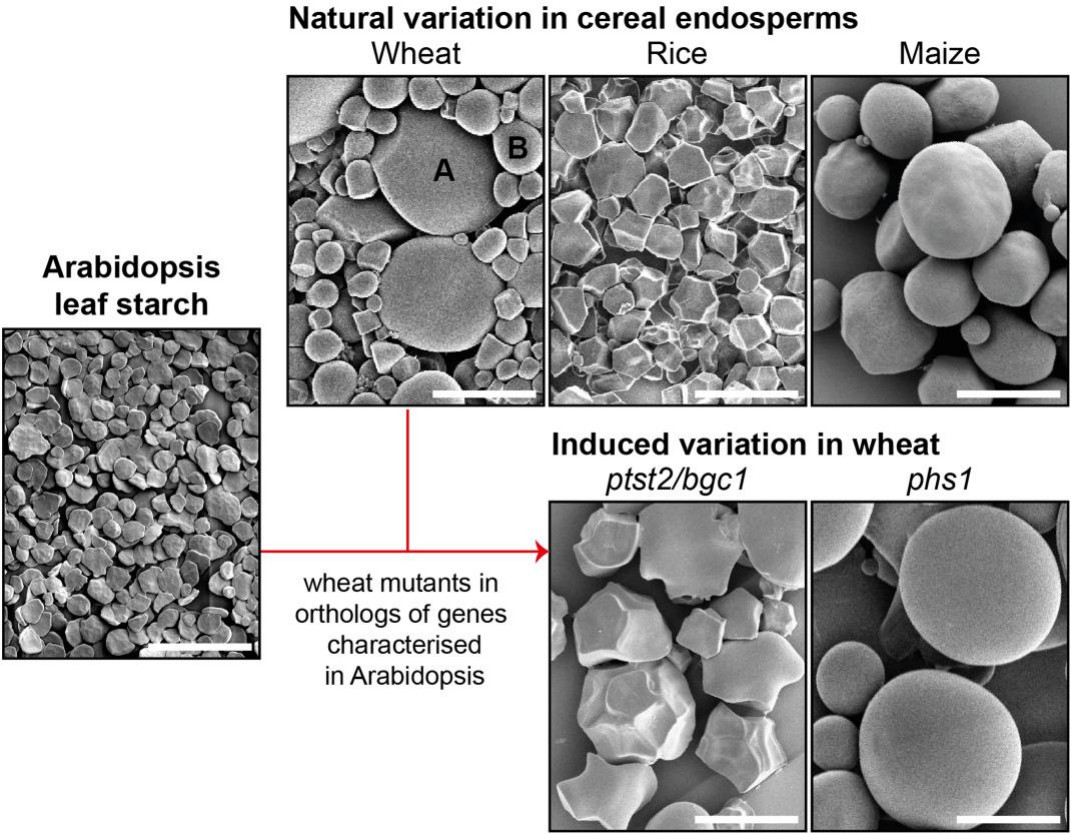
Starch granule morphology observed using scanning electron microscopy. Arabidopsis produces starch in its leaves, and the starch granules are relatively small and have a flattened appearance. In the endosperm starch of cereals, there is vast natural variation in granule shape and size. Wheat starch has distinctive bimodal granules that can be further classed into large A- and small B-type starch granules (marked in the figure). Variation in granule morphology can be induced in wheat by mutating granule initiation genes characterized in Arabidopsis. Bars = 20 *µ*m.

The main challenge in translating findings between Arabidopsis and crops is the enormous natural variation between plant species and organs in starch structure (Matsushima 2015; Tetlow and Emes 2017) and the metabolic context in which the starch is synthesized (Smith and Zeeman 2020). Starch is produced in plastids as semi-crystalline, insoluble starch granules, which are composed of 2 different glucose polymers—amylopectin and amylose (Seung 2020). Arabidopsis, like most other plants, produces starch primarily in the photosynthetic chloroplasts of leaves and degrades it at night to sustain metabolism and growth (Graf et al. 2010). Multiple starch granules accumulate between the thylakoid membranes of the chloroplast. This is distinct from most crop organs, where starch is synthesized in nonphotosynthetic amyloplasts continuously through grain or storage organ development. There is also massive variation in starch granule morphology between species (Fig. 4).

Nevertheless, Arabidopsis has been essential for discovering new players involved in starch synthesis, and almost all these players are now known to also play important roles in various crops. However, the exact mechanisms by which they act differs depending on species and organs.

There are many examples to illustrate this, but I will highlight the recent advances in our understanding of starch granule initiation (Seung and Smith 2019). Although the biochemical reactions required for amylopectin and amylose synthesis were relatively well understood, the steps required for initiating the synthesis of a starch granule were unknown. Almost a decade ago, Sam Zeeman (ETH Zurich) and I characterized in Arabidopsis the PROTEIN TARGETING TO STARCH (PTST) family of proteins, which are glucan-binding proteins that interact with other biosynthesis enzymes, presumably assisting with substrate binding (Seung et al. 2015, 2017). One member of this family is PTST2, which plays an important role in granule initiation. Instead of having multiple granules per chloroplast, Arabidopsis mutants deficient in PTST2 typically have only 1 large starch granule per chloroplast (Seung et al. 2017). We proposed that PTST2 acts in granule initiation by interacting with STARCH SYNTHASE 4 (SS4), which was known to be the major enzyme involved in granule initiation (Roldán et al. 2007).

The discovery of PTST2 led us to identify 4 other non-enzymatic proteins that interact with PTST2 and play important roles in granule initiation (Seung et al. 2018; Abt et al. 2020). The rapid pace at which we discovered this suite of proteins was largely due to the ease of genetic and biochemical approaches in Arabidopsis.

When I started at the John Innes Centre, we aimed to understand starch granule initiation in developing wheat grains. Wheat endosperm starch is inherently different from Arabidopsis leaf starch in that it has 2 distinct types of starch granules—large A-type granules and small B-type granules (Fig. 4). A-type granules form during early grain development, while B-type granules are initiated around 10 to 15 days after the A-type granules. The role of the wheat PTST2 ortholog (referred to as B-GRANULE CONTENT 1, BGC1) on these 2 distinct waves of granule initiation could not have been predicted from results in Arabidopsis. Firstly, both PTST2/BGC1 and SS4 play important roles in the initiation of both A-type granules (Chia et al. 2020; Hawkins et al. 2021). In wheat mutants lacking either protein, most amyloplasts initiated many smaller granules during early grain development rather than a single A-type granule. The smaller granules grew up against each other to form “compound” granules—where individual granules appear polygonal due to impaction; this is a type of granule naturally found in other cereals (such as rice) (Matsushima 2015). This was consistent with findings in Arabidopsis, which showed that these proteins are important for controlling starch granule number. However, it was essentially the opposite phenotype of the Arabidopsis mutants (Roldán et al. 2007; Seung et al. 2017), with more rather than fewer granules per plastid.

However, PTST2/BGC1 is also important for B-type starch granule initiation, because reduced function in this gene leads to fewer B-type granules—a discovery that was led by Kay Trafford's team (Chia et al. 2020). Interestingly, we later discovered that BGC1 acts with another enzyme, the plastidial glucan phosphorylase (PHS1), and not SS4 in B-type granule initiation (Kamble et al. 2023). Knockout mutants in *PHS1* in wheat had significantly fewer B-type granules (Fig. 4). In contrast, Arabidopsis *phs1* mutants have no effect on starch granule number (Malinova et al. 2017). This suggests that A- and B-type granule initiations occur through distinct biochemical mechanisms, a distinction that could not have been determined solely using Arabidopsis.

Arabidopsis therefore gave us a head start in developing our current model of starch granule initiation in wheat grains by providing candidate genes to initially study. However, further research in wheat was essential to discover how the functions of those genes mediate the distinct temporal pattern of A- and B-type granule initiations. We believe we will discover many more examples of functional diversification in granule initiation proteins of other species that have different initiation patterns compared with wheat. We already know that knocking out *PTST2* orthologs in rice and *Brachypodium* has effects on granule initiation that are distinct from that in wheat (Peng et al. 2014; Watson-Lazowski et al. 2022; Yan et al. 2024). Characterizing these differences will lead to mechanisms underpinning interspecies diversity in granule morphology. For now, we can already manipulate starch granule shape and size in wheat using the components we characterized (Fig. 4) (Chen et al. 2021). We are now working with food researchers and industrial partners to examine how altering starch granule morphology can benefit bread- and pasta-making quality, nutrition, and gut health.

## Beyond face value—understanding and engineering plant surfaces

(Written by Linsan Liu and Sarah M. McKim)

Land plants are equipped with exquisite epidermal adaptations to cope with the aerial environment. The outer epidermal cell layer, consisting mostly of pavement cells, prevents surface water loss by secreting an external, impermeable waxy cuticle (González-Valenzuela et al. 2023). Gas exchange and transpiration occur instead through stomata, regularly spaced air pores that open and close via movement of 2 opposing guard cells (Lawson and Morison 2004). As plants expanded into diverse terrestrial niches, from arid plains to high-altitude plateaus, they often evolved further adaptive epidermal elaborations, including novel cell types, different stomatal morphologies, and cuticular specializations (Koch et al. 2008). Accordingly, exploiting epidermal variation in crops could stabilize and improve yield under changing climates; for example, less permeable cuticles coupled with faster, fewer stomata may help cereals perform better in warmer, drier conditions (Jolliffe et al. 2023). However, we know relatively little about epidermal features in crops in contrast to the super-tractable but non-crop Arabidopsis, where study of cuticle mutants, especially the *eceriferum* (*cer*, “waxless”) alleles, as well as disrupted stomatal patterning mutants has identified genes, metabolic pathways, and processes important for cuticle and stomata formation (Lee and Bergmann 2019; Lee and Suh 2022). Perhaps reflecting their deep adaptive importance, many of these genes appear broadly conserved and represent fortunate, low-hanging translational fruit, such as in cases where transgenic overexpression of orthologous cuticular genes increased disease and drought resiliency in multiple crops (Kong et al. 2020; Liu et al. 2022b). However, unique cuticular chemistries and novel epidermal cell types in crops along with gene subfunctionalizations mean that the Arabidopsis model is insufficient to predictively engineer all epidermal traits important for crop performance (Kong et al. 2020).

**Figure 5.**
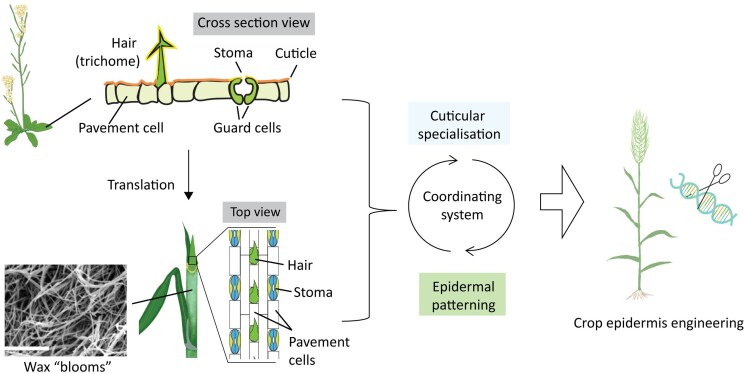
Complementary research in Arabidopsis and crops aids understanding of epidermal features in plants and the engineering of plant surfaces for improved crop performance. The outer epidermal cell layer is covered by cuticle and made up of mostly pavement cells interspersed with other specialized cell types. In Arabidopsis, specialized epidermal cells are scattered across the leaf surface, while in cereal grasses such as barley, epidermal cells are arranged with pavement cells in distinctive files. In both cases, specialized cells are spaced away from each other. Cuticles can vary by cell type and also show specializations between species, such as crystalline wax blooms in barley. Research on Arabidopsis greatly informs our understanding of genes and pathways guiding epidermal cell spacing and cuticular metabolism in crops and together reveals extensive conservation but also variation and novelty across species. Excitingly, complementary research in both Arabidopsis and grass models paint an emerging picture of how the regulation of cuticular specializations and epidermal patterning may be coordinated. Leveraging these insights will help us better engineer crops for desired epidermal traits. SEM photos present wax blooms on the leaf sheath of barley. Scale bar = 5 *µ*m.

We argue that fundamental research in crops compliments work in Arabidopsis to bridge this knowledge gap and enhances our understanding plant surface evolution. Our recent investigation of barley *cer* mutants lacking the “wax bloom,” a late-stage, bluish-white (glaucous) cuticular deposition associated with increased yield, nicely showcases this beneficial interaction (Fig. 5). In contrast to the typical plant cuticular waxes made up of very long chain fatty acids (VLCFAs) and their derivatives (González-Valenzuela et al. 2023), the wheat and barley wax bloom is dominated by β-diketones and OH-β-diketones cuticular crystals absent in Arabidopsis. Consequently, exploiting cereal wax bloom mutants was essential to identify the novel metabolic gene cluster responsible for β-diketone and OH-β-diketone synthesis (Hen-Avivi et al. 2016; Schneider et al. 2016). Nonetheless, our study of the barley wax bloom mutant *cer-x* identified the first upstream regulator of the bloom and metabolic cluster expression as HvWAX-INDUCER1 (HvWIN1) (McAllister et al. 2022), a barley ortholog of the WIN/SHINE (SHN) transcription factors originally characterized in Arabidopsis to promote leaf VLCFAs (Aharoni et al. 2004). Interestingly, *HvWIN1* appears dispensable for barley leaf VLCFAs (Aharoni et al. 2004), highlighting how conserved transcriptional regulators target different cuticular components across species. Moreover, the other SHN transcription factor in barley, NUDUM (NUD), appears further unfunctionalized to promote grain-to-hull adhesion, a protective adaption unique to *Hordeum* ssp. and beneficial in malting (Taketa et al. 2008). Intriguingly, we noticed that multiple *cer* mutants also showed defective hull adhesion. We have cloned several of these genes that appear to act both dependent and independent of NUD, with 1 published so far (Campoli et al. 2024). In all cases, their molecular function was informed by their previous characterization in Arabidopsis and collectively represent an example of a crop-specific cuticular specialization (hull adhesion) driven by repurposed epidermal regulatory pathways.

In addition to hull adhesion, wax bloom mutants have also been associated with defective epidermal patterning on leaves (Zeiger and Stebbins 1972; Gray et al. 2000). Understanding this pleiotropy is part of a long-standing mystery to explain why mutants in cuticle formation influence epidermal cell patterning and vice versa across plants (Bird and Gray 2003; Berhin et al. 2022). Excitingly, our recent work and that of others point to an underlying circuitry involving stomatal patterning (Liu et al. 2022a; Yang et al. 2022; Zhang et al. 2022). Despite the difference in epidermal cell organization between Arabidopsis, where stomata form in dispersed locations in the expanding leaf, and grasses, where strict parallel cell files with specific lineages, including stomata, are formed much earlier, both show an alternating pavement cell to stomata arrangement important for proper stomatal movement (Fig. 5). Best understood in Arabidopsis, this pattern originates from an asymmetric cell division (ACD) that generates a smaller daughter cell expressing core stomatal factors that divides again to form the pore, and a larger daughter cell, where signaling cascades downstream of EPIDERMAL PATTERNING FACTOR (EPF) peptides degrade these stomatal factors, promoting direct differentiation into a pavement cell (Lee and Bergmann 2019). Many components of this pathway are conserved between Arabidopsis and other plants, with overexpression of native and even synthetic EPFs reducing stomatal density and improving drought tolerance in multiple cereals (Hughes et al. 2017; Caine et al. 2019; Ferguson et al. 2024; Karavolias et al. 2024). In fact, we showed that 2 barley mutants, initially described for their patchy wax blooms, *cer-g* and *cer-s*, are defective alleles of inhibitory cascade components that cause stomatal “clusters” when the larger daughter cell divides at least 1 more time and forms stomata rather than pavement cells (Liu et al. 2022a), consistent with the phenotypes of loss-of-function mutants in these *cer* gene orthologs in Arabidopsis. However, we also revealed that these mutants show more permeable cuticles and substantial reductions in cuticle-related gene expression during these early patterning events, suggesting that these inhibitory cascades also promote cuticle deposition as well as pavement cell fate (Liu et al. 2022a). Our work adds to accumulating evidence from Arabidopsis that dynamic tuning of cuticle properties, often with deposition promoted by EPF-driven cascades and repressed by core stomatal factors, is important for asymmetric cell fate and stomatal progression (Gray et al. 2000; Smit et al. 2023) with possible roles in environmental control of stomatal density (Kaufman et al. 1985).

This type of regulatory interplay may be relevant to the “improved” surfaces from overexpression of cuticular regulators in cereals that may also impact stomatal density but also to other epidermal features in grasses. Grasses develop additional epidermal file lineages, including those for distinctive prickle cells, shrinking bulliform cells, and rigid silica cells (sometimes with accessory cork cells), which, while less studied, are all likely adaptive in arid conditions (Kaufman et al. 1985; Werker 2000; Matschi et al. 2020). Using grass models to understand these specialisms often finds repurposing stomatal and other patterning systems first characterized in Arabidopsis (Raissig et al. 2017; Abrash et al. 2018; Schuler et al. 2018; Sun et al. 2020). In fact, we noted that *cer-g* and *cer-s* barley mutants show clustered prickle hairs as well as clustered and weakened identity silica/cork cell pairs, cells whose spacing also derive from ACDs. Moreover, these mutants also showed switching of specialized cell fate could switch within a single file. Taken together, these phenotypes suggest that these pathways promote asymmetric cell fate in multiple file types but also reinforce file identity, thus playing a pivotal patterning role across the entire epidermis.

Understanding the higher order of file placement as well as genetic and functional interactions between epidermal traits are key problems in grass epidermal research. However, existing mutant resources offer an excellent genetic handle—rescreening in our laboratory has revealed more wax bloom mutants with substantial epidermal patterning changes, including roles for HvWIN1, suggesting that easily observable cuticle defects may be a proxy for defective epidermal patterning (personal observation) and a platform to explore possible mechanistic relationships between early patterning decisions about cell fate and later appearing epidermal features like the wax bloom. In our current work, we aim to integrate precise genetic, transcriptomic, chemical, and ultrastructural analyses to unravel the hierarchy coordinating grass epidermal features.

In summary, combined insights from both Arabidopsis and grass models work together towards a comprehensive understanding of epidermal development and possible routes to uncouple pleiotropic effects (Fig. 5). By leveraging their respective strengths and conducting comparative research, we will further enhance our genetic and molecular toolkits for engineering this critical adaptive feature of plants, aiding in the development of crops with improved productivity and resilience under current and future climates.

## Arabidopsis research into the future

(Written by all authors)

Arabidopsis remains an invaluable model system not only for developing and refining technologies but also as an in planta platform for validating biochemical and metabolic pathways. The increasing availability of genomic data and advanced transformation and genome-editing tools in crops has expanded the potential for functional genetics and molecular studies directly in these species, leading to a shift away from exclusive reliance on Arabidopsis. However, this molecular progress has also reinforced the need for fast and tractable model systems to further characterize mechanisms identified in crops. With its ease of growth, efficient transformation, minimal growth-space requirements, and well-established resources, Arabidopsis should be considered as a key component of the crop researcher's toolbox, alongside systems like *Nicotiana benthamiana*, for experiments such as complementation assays, protein localization, and protein-protein interaction studies. Moreover, many fundamental biological processes, including those discussed here, remain incompletely understood in Arabidopsis itself, ensuring its continued role as a source of novel genes and mechanisms relevant to crops. Finally, Arabidopsis serves as a critical reference for understanding the evolution of pathways and traits. A growing trend in plant sciences is to explore how plants differ rather than assuming uniformity based on a single model species. The extensive knowledge generated from Arabidopsis provides an essential framework for comparative studies, helping to distinguish conserved mechanisms from lineage-specific innovations.

## Data Availability

No new data were generated or analysed in support of this review.

## Dive Curated Terms

The following phenotypic, genotypic, and functional terms are of significance to the work described in this paper:

PHS1 Gramene: At3g29320
PHS1 Araport: At3g29320

starch CHEBI: CHEBI:28017

cytochrome CHEBI: CHEBI:4056

PTST2 Gramene: AT1G27070
PTST2 Araport: AT1G27070
